# Commensal Homeostasis of Gut Microbiota-Host for the Impact of Obesity

**DOI:** 10.3389/fphys.2017.01122

**Published:** 2018-01-08

**Authors:** Pengyi Zhang, Xiangjing Meng, Dongmei Li, Richard Calderone, Dewei Mao, Bo Sui

**Affiliations:** ^1^Sport Science Research Center, Shandong Sport University, Jinan, China; ^2^Department of Microbiology & Immunology, Georgetown University Medical Center, Washington, DC, United States; ^3^Shandong Academy of Pharmaceutical Science, Jinan, China

**Keywords:** gut microbiota, enterotypes, interactions, commensal homeostasis, obesity, diet

## Abstract

Gut microbiota and their metabolites have been linked to a series of chronic diseases such as obesity and other metabolic dysfunctions. Obesity is an increasingly serious international health issue that may lead to a risk of insulin resistance and other metabolic diseases. The relationship between gut microbiota and the host is both interdependent and relatively independent. In this review, the causality of gut microbiota and its role in the pathogenesis and intervention of obesity is comprehensively presented to include human genotype, enterotypes, interactions of gut microbiota with the host, microbial metabolites, and energy homeostasis all of which may be influenced by dietary nutrition. Diet can enhance, inhibit, or even change the composition and functions of the gut microbiota. The metabolites they produce depend upon the dietary substrates provided, some of which have indispensable functions for the host. Therefore, diet is a key factor that maintains or not a healthy commensal relationship. In addition, the specific genotype of the host may impact the phylogenetic compositions of gut microbiota through the production of host metabolites. The commensal homeostasis of gut microbiota is favored by a balance of microbial composition, metabolites, and energy. Ultimately the desired commensal relationship is one of mutual support. This article analyzes the clues that result in patterns of commensal homeostasis. A deeper understanding of these interactions is beneficial for developing effective prevention, diagnosis, and personalized therapeutic strategies to combat obesity and other metabolic diseases. The idea we discuss is meant to improve human health by shaping or modulating the beneficial gut microbiota.

## Introduction

Gut microbiota is identified as a relatively new and key player in the treatment of obesity. Changes in the gut microbiota, have been shown to not only correlate with good health but, conversely, lead to the pathogenesis of obesity and various metabolic diseases. It is hoped that a defined composition of gut microbiota can prevent or even cure obesity and related diseases. Interactions of the gut microbiota and host, or metabolites they produce, are still under investigation. Gut microbiota represents a large number and complex community of microorganisms and their genetic material living in the intestines of humans and other animals. The gut microbiota has been linked with a number of chronic diseases such as obesity, diabetes, and other metabolic diseases (Zhao, 2013). As known, obesity is an increasingly serious international health issue that can increase the risk of the insulin resistance and other metabolic syndromes (Frayling et al., 2007; Perry et al., 2016). In this review, the causality between the composition of the gut microbiota and its role in the pathogenesis of obesity and obesity-related metabolic disorders are critically described, based on metagenome analysis of the microbiota, microbe-microbe, and microbiota-host interactions, and the specific metabolites produced by microbiota and host (Musso et al., 2010).

## Homeostasis of enterotype and host

The gut microbiota has a collective metabolic activity, which impacts and responds to the host as an integrated virtual organ. Some components of the “organ” can be negative factors and contribute to the pathogenesis of various metabolic diseases. The adverse effect can be reversed by modulating one's diet to build a helpful commensal community. The mechanisms that the gut microbiota has on metabolic homeostasis and immune responses are currently being unraveled (O'Hara and Shanahan, 2006). Depending on the species and functional composition of the human gut microbiota, the host can be classified as enterotypes (species) which represent a highly aggregated microbial community structure in multidimensional space (Arumugam et al., 2011; Ding and Schloss, 2014). Based on the proportion of genera/species, their abundance, specific metabolites, and collective functions, the phylogenetic compositions of gut microbiota from individuals living in these environments have been analyzed. The results indicate that the gut microbiota can be mainly arranged as four enterotypes: *Bacteroides, Prevotella, Rminococcus*, and *Firmicutes* (Gill et al., 2006; Kurokawa et al., 2007; Zoetendal et al., 2008; Jensen et al., 2009). The enterotypes have different dominant classifications, pathways, functions, and correlations in abundance of co-occurring genera. The enterotype of *Bacteroides* has the potential to metabolize carbohydrates and proteins through the enzymes involved in glycolysis and pentose phosphate pathways (Martens et al., 2009). Enterotype *Prevotella* is able to act on the gut mucin oligosaccharide synergistically (Wright et al., 2000). In spite of the degradation of mucin, enterotype *Ruminococcus* also promotes the transport and uptake of monosaccharides by enriching in the membrane and binding mucin for hydrolysis (Derrien et al., 2004). Enterotype *Firmicutes* interacts positively with fiber but negatively with fat (Wu et al., 2011). The enterotypes use different strategies to acquire energy from the substrates available in the gut ecosystem. The specific compositions of enterotypes respond to the special metabolic mechanisms for carbohydrate, amino acids, and fatty acid metabolism. These strategies determine the incidence of obesity and related metabolic diseases (Devaraj et al., 2013). Thus, the gut microbiota provides an important contribution to the health status of individuals. The studies of enterotypes could be utilized to assess and diagnose the numerous human metabolic disorder syndromes, for instance obesity and complications such as diabetes and cardiovascular pathologies.

The formation of enterotypes is closely related to individual long-term diets or drug intake. Even for short-term changes in the microbiota, the enterotypes can still remain relatively stable. But the human gut microbiota can change rapidly, for example, within one day to cope with the drastic changes in diet and medical therapies as a result of disease intervention (Wu et al., 2011). A high-fat diet could promote changes in the gut microbiota composition, as described with the replacement of the *Bacteroidetes* enterotype by both *Firmicutes* and *Proteobacteria* (Hildebrandt et al., 2009). These types of changes are characteristic of a well-balanced host-microbial symbiosis due to diet and responses to medications. Understanding the roles of particular enterotypes and changes will be helpful to an intervention in the pathogenesis of obesity and related diseases and to personalize therapies.

## Homestasis of gut microbiota-host interactions

The diversity and specificity of human gut microbiota closely correlated to the host genotype, diet, and metabolites mediating this interaction (Nicholson et al., 2012). The host genotype can impact the physical status of the human body by regulating the gut microbiota. Metagenome analysis of gut microbiota in lean and obese individuals indicated significant differences in genotype and its richness. It allowed these investigators to analyze a few bacterial markers to distinguish the level of individual microbial species and genotype richness (Le Chatelier et al., 2013). In turn, the genotype richness could predict the risk of obesity, our understanding of the commensal relationship, and identify potential therapeutic targets (Goodrich et al., 2014). One species of a little-known intestinal bacterium was identified through a study of twins. It is highly inheritable and more common and personified in slender people. In this study, 1,000 fecal samples were collected from 416 pairs of twins and the microorganisms were identified by gene sequencing. Compared to the dizygotic twins, the gut microbiota of monozygotic twins who have the complete same genome is more concordant, especially in the family *Christensenellaceae* which was the most heritable taxon and enriched in individuals with low body mass index (BMI). A similar observation of weight loss was noted in fecal transplanted, germ-free mice (Goodrich et al., 2014). Thus, the human genome has a profound effect on the composition of gut microbiota. Increasing the abundance of *Christensenellaceae* could help individuals prevent obesity. The gut microbiota just described would be a new approach for obesity treatment. Personalized therapy with probiotics could be built on the foundation of individual genomes. According to this study, these specific intestinal bacteria are inheritable, and their diversity mainly depends on the host genotype but not other environment impact factors. The same result was also found in the gut microbiota transplanted experiment from four pairs of obese and lean human twins to germ-free mice respectively (Ridaura et al., 2013; Cox et al., 2014). Mice transplanted with microbiota from the obese one of the twins also exhibited an increase in weight and adiposity despite given the same diet. Interestingly, obesity was reduced when the obese and lean mice lived together to share and change their gut microbiomes.

Furthermore, diet modulates the activity of host and gut microbiota synchronously to influence their interaction. A high-fat diet enhanced gene expressions of both the host and microbiota. The genes whose expression increased included a colonic goblet, cell-specific gene (RELMβ) of the host, and genes for bacterial chemotaxis and flagellar assembly in the gut microbiota (Hildebrandt et al., 2009). In the obese mice, a high triglyceride-derived fatty acid intake was commonly accompanied with hyperexpression of the enzymes that digest polysaccharides and lipoproteins by the gut microbiota (Bäckhed et al., 2004). *Bacteroides thetaiotaomicron* is identified as an emerging obesity-associated gut microbial species. It alleviated diet-induced body-weight gain and adiposity by altering circulating amino acids (Liu et al., 2017). The gut microbiota and host could communicate with each other by the universal language “eukaryotic signaling molecule.” Thus, *N*-acyl amides produced by gut microbiota could interact with GPR119, G-protein coupled receptor (GPCR), to regulate metabolic hormones and glucose homeostasis (Cohen et al., 2017). Perhaps GPR119 might be the potential target molecule in the treatment of obesity and diabetes.

Interestingly, germ-free mice or mice treated with antibiotics to reduce the gut microbiome showed a better glucose tolerance and insulin sensitivity, and could maintain a low BMI. The germ-free mice had less food intake and more energy expenditure than normal mice (Turnbaugh et al., 2006). Even under a high-fat diet, the germ-free mice remained slim and also produced more beige fat when the normal mice become obese (Suárez-Zamorano et al., 2015). Meanwhile, antibiotic treatment in early life changed the gut microbial composition and the activity of genes associated with carbohydrate and lipid metabolism, as well as the level of special hormones, leading to the obesity (Cho et al., 2012). The MHC-II-mediated protection from type I diabetes was significantly affected due to the destruction of gut microbiota homeostasis (Silverman et al., 2017). Therefore, short-term and low doses of antibiotics had a long-term effect on young rats, altering the microbiome-host metabolic interactions, leading to the obesity in their middle age. These data suggest that the gut microbiota in the early stages of life may affect the formation of metabolic signaling pathways in the host (Cox et al., 2014).

## Exogenous and endogenous effectors to dynamic balance of gut microbiota

The gut microbiota is able to modulate multiple host metabolic pathways, interactive host-microbiota metabolism and signal transmission, and microbiome-gut-brain axis reactions (Nicholson et al., 2012). The dietary fiber-derived short chain fatty acids (SCFAs) and their receptors are recognized as one set of important mediator links of diet to gut microbiota-host homeostasis (Turnbaugh et al., 2006). SCFAs activate GPR41 and GPR43, two SCFA-specific GPCRs, to induce satiety and energy expenditure by promoting the secretion of the gut hormone peptide YY (PYY), and induce insulin secretion and adiposity reduction in obese individuals by significantly enhancing PYY and glucagon-like peptide-1 (GLP-1) secretion, respectively (Samuel et al., 2008; Thomas et al., 2009; Tolhurst et al., 2012; Chambers et al., 2015; Li et al., 2017). Especially, the acetate was shown to be the key factor leading to obesity. An altered gut microbiota increased the production of acetate in rats, which increased the risk of the activation of the parasympathetic nervous system, promoted pancreatic β cells to increase glucose-stimulated insulin secretion (GSIS), triggered ghrelin secretion, led to hyperphagia, obesity, insulin resistance, and other related syndromes. Finally, the complete process of rodent obesity caused by the gut microbiota disbalance was established. The results demonstrated increased acetate production due to the nutrient-gut microbiota and the subsequent parasympathetic activation could be developed as potential therapeutic targets for obesity (Perry et al., 2016). SCFAs and correlate cascade signal pathways unraveled a novel strategy to achieve the gut microbiota-host commensal homeostasis. Simultaneously, this efficient dietary intervention is also conducive to create a well-developed therapy and to prevent obesity and energy metabolic syndromes (Khan et al., 2014). Meanwhile, a recent study showed that omega-3 fatty acids were closely related to gut microbiota diversity. It also reduced the oxidative stress of intestines by inducing the production of N-carbamylglutamate (NCG) (Menni et al., 2017). In addition to the exogenous effectors described above, a α-MSH mimetic bacterial protein ClpB identified as an endogenous effector from *E. coli* K12 during exponential growth, was involved in the gut microbiota-brain signal pathway. By promoting PYY and GLP-1 secretion, activating c-Fos in hypothalamic proopiomelanocortin (POMC) neurons, the host reduced hunger and increased satiety. Thus, the mediator expressed during the nutrient-induced *E. coli* growth could be used as the signal to terminate eating since it had an impact on the diet process (Breton et al., 2016).

In the patients with severe disbalance of gut microbiota due to antibiotic treatment, prebiotics and probiotics were heavily consumed and significantly decreased. Short-term, randomized controlled trials showed that prebiotics and probiotics were beneficial to insulin sensitivity, postprandial incretins, and glucose tolerance (Musso et al., 2010). Prebiotics, inulin-typefructans (ITF), and arabinoxylan-oligosaccharides (AXOS) restored the balance of gut microbiota and increased the number of *Bifidobacteria* and butyrate-producing colon bacteria (Broekaert et al., 2011). The proportion of the latter in the intestinal microbiome was a key marker in the protection against obesity and type II diabetes, which indicated that the structure of the intestinal microbial community can be used as an effective environmental factor to prevent and diagnose these chronic metabolic diseases. Based on the metagenome analysis, different intestinal microbiome markers were screened and selected to establish the prediction model of obesity and type II diabetes for Chinese and Europeans, respectively (Qin et al., 2012; Karlsson et al., 2013; Cai et al., 2015; Rivière et al., 2016). A return to a healthy state occurs when the proportion of “hunger” microbes and the proportion of microbes that cause “satiety” in the gut microbiota are in a dynamic equilibrium state (Rooks and Garrett, 2016). A deeper understanding of these functions will be beneficial for establishing effective therapeutic strategies to combat obesity and correlate diseases, and improve health by modulating the gut microbiota.

## Energy homeostasis under diet intervention

The prevalence of obesity is tightly linked to excessive energy availability and sedentariness. The gut microbiota could induce or modulate the signal transmission directly or indirectly to affect energy homeostasis (Rosenbaum et al., 2015). The quali-/quantitative changes in gut microbiota composition affect both energy balance (intake and expenditure) and energy storage which would lead to the development of obesity in the dysfunctional state (Scarpellini et al., 2010; Kobyliak et al., 2016). The obese gut microbiota changes the abundance of the two dominant enterotypes, the *Bacteroidetes* and the *Firmicutes*, to gain more energy production (Turnbaugh et al., 2006). This study which was carried out in the adult female monozygotic and dizygotic twins, and their mothers, indicated that the gut microbiota could be shared among the family members, but with variation among individuals. Some of the metabolites generated by enterotypes closely correlate with energy acquisition and subsequently lead to a risk of obesity in the host. These enterotypes enhanced the efficiency of energy extraction and fat storage via enzymes related to fermentation (Tilg and Kaser, 2011). Germ-free mice fed different combinations of saturated fat and high-fiber food revealed a transmissible, rapid, and modifiable effect of diet by gut microbiota (Turnbaugh et al., 2009; Ridaura et al., 2013). The investigation of diet intervention conducted in the obese and overweight individuals found that the abundance of gut microbiota was increased by high-fiber and low-fat diet, which improved the clinical symptoms associated with obesity (Cotillard et al., 2013). The Life Lines-DEEP project analyzed the relationship of the gut microbiota and 126 exogenous and intrinsic host factors, including 60 dietary factors, 18.7% of which was associated with the variation in human gut microbiota composition (Zhernakova et al., 2016). A group of “core microbiome” genes was identified and shared among 154 individuals, which was associated with obesity at phylum-level changes. The obesity reversely reduced the microbial diversity, altered the gene expression and metabolic pathways and ultimately the energy harvest (Turnbaugh and Gordon, 2009). The Flemish Gut Flora Project analyzed the effect of various factors on the intestinal microbial diversity. It revealed a 14-genera core microbiota and sixty-nine covariates associated to microbiota variation (Falony et al., 2016). In addition, the association of microbial communities and dietary patterns was also elucidated by use of a mathematical model in clinical trials, which tried to create the universal rules of the molecular mechanism of intestinal microbial interaction and predict the responses of different patients to the modified diet (Qin et al., 2010). Compared to the host genotype, diet played a more important role on the variation of the individual gut microbiota, which showed a definite dose-dependent relationship with the response to diet. Subtle changes in diet might cause changes in gut microbiota compositions (Carmody et al., 2015).

## Conclusion

**Figure 1:**
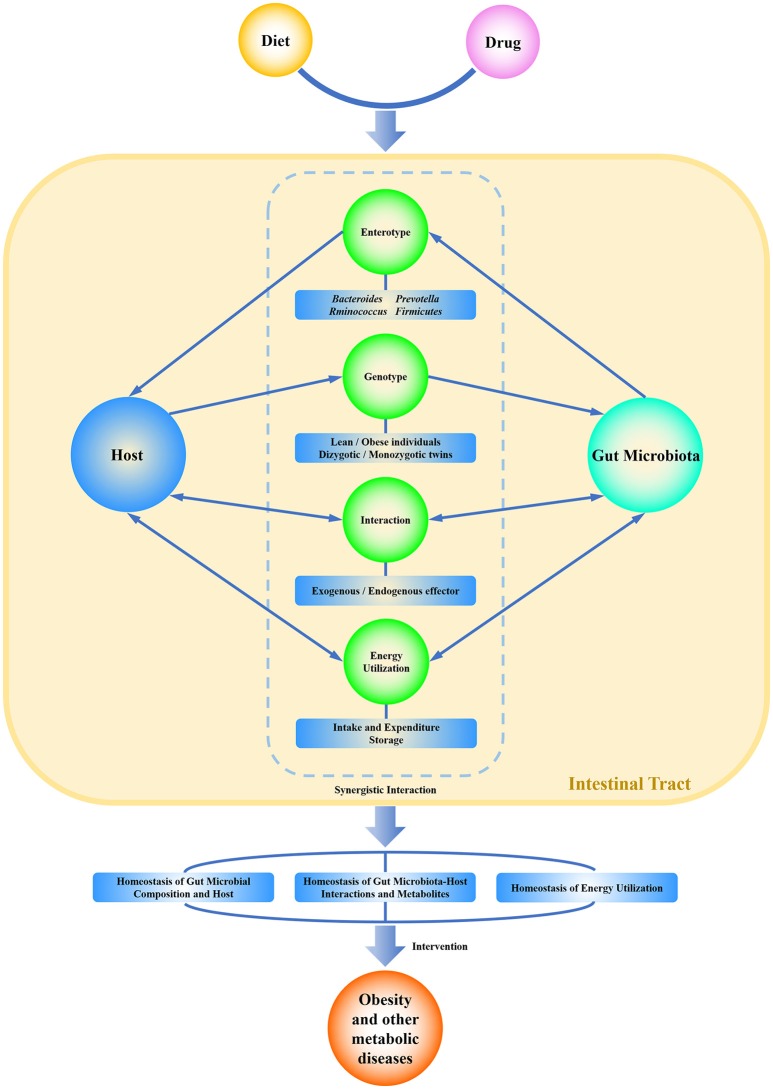
Graphical representation of the commensal homeostasis of gut microbiota-host under the intervention of diet and drugs. Diet and drugs can be digested, absorbed, and metabolized by the host and gut microbiota. The host and gut microbiota can interact synergistically to impact the physiological status of the human body. They not only support themselves directly by using nutritional substrates but also impact each other indirectly by enterotype, genotype, metabolites, and related functions. Ultimately, the host and gut microbiota achieve a commensal homeostasis of composition, interactions, metabolites, and energy utilization.

The focus of this review was developed from research on the structure and function of gut microbiota as well as the integration of gut microbiota-host interactions at a higher dimension. The commensal pattern of gut microbiota will be made clearer through gene sequencing, metagenome analysis, gut microbiota-host responses, and verification in gnotobiotic animals. The diet intake and the specific metabolites produced by the host and the microbiota can be utilized to change or recover the ecosystem of intestinal homeostasis either in health or pathologic conditions (Scarpellini et al., 2010). Modulation of gut microbiota could be a useful and alternative method to block and even cure obesity and other metabolic syndromes. A healthy lifestyle, including the reasonable composition of dietary nutrition and the avoidance of excessive energy intake, may establish a friendly gut microbiota-host homeostasis. The commensal homeostasis of gut microbiota is favored by a balance of microbial composition, metabolites, and energy (Figure 1). In turn, this ideal relationship may have positive effects on the prevention and therapy of obesity and other related metabolic diseases.

## Author contributions

PZ and XM: contributed to conception, design and manuscript writing; DL, RC, DM, and BS: all contributed substantially to the writing and revision of the manuscript and approved its final version.

### Conflict of interest statement

The authors declare that the research was conducted in the absence of any commercial or financial relationships that could be construed as a potential conflict of interest. The reviewer LW and handling Editor declared their shared affiliation.

## References

[B1] Arumugam M. Raes J. Pelletier E. Le Paslier D. Yamada T. Mende D. R.. (2011). Enterotypes of the human gut microbiome. Nature 473, 174–180. 10.1038/nature09944 21508958 PMC3728647

[B2] Bäckhed F. Ding H. Wang T. Hooper L. V. Koh G. Y. Nagy A.. (2004). The gut microbiota as an environmental factor that regulates fat storage. Proc. Natl. Acad. Sci. U.S.A. 101, 15718–15723. 10.1073/pnas.0407076101 15505215 PMC524219

[B3] Breton J. Tennoune N. Lucas N. Francois M. Legrand R. Jacquemot J.. (2016). Gut commensal, *E. coli* proteins activate host satiety pathways following nutrient-induced bacterial growth. Cell Metab. 23, 324–334. 10.1016/j.cmet.2015.10.017 26621107

[B4] Broekaert W. F. Courtin C. M. Verbeke K. Van de Wiele T. Verstraete W. Delcour J. A. (2011). Prebiotic and other health-related effects of cereal-derived arabinoxylans, arabinoxylan-oligosaccharides, and xylooligosaccharides. Crit. Rev. Food Sci. Nutr. 51, 178–194. 10.1080/10408390903044768 21328111

[B5] Cai L. Wu H. Li D. Zhou K. Zou F. (2015). Type 2 diabetes biomarkers of human gut microbiota selected via iterative sure independent screening method. PLoS ONE 10:e0140827. 10.1371/journal.pone.0140827 26479726 PMC4610706

[B6] Carmody R. N. Gerber G. K. Luevano J. J. Gatti D. M. Somes L. Svenson K. L.. (2015). Diet dominates host genotype in shaping the murine gut microbiota. Cell Host Microbe 17, 72–84. 10.1016/j.chom.2014.11.010 25532804 PMC4297240

[B7] Chambers E. S. Viardot A. Psichas A. Morrison D. J. Murphy K. G. Zac-Varghese S. E.. (2015). Effects of targeted delivery of propionate to the human colon on appetite regulation, body weight maintenance and adiposity in overweight adults. Gut 64, 1744–1754. 10.1136/gutjnl-2014-307913 25500202 PMC4680171

[B8] Cho I. Yamanishi S. Cox L. Methé B. A. Zavadil J. Li K.. (2012). Antibiotics in early life alter the murine colonic microbiome and adiposity. Nature 488, 621–626. 10.1038/nature11400 22914093 PMC3553221

[B9] Cohen L. J. Esterhazy D. Kim S. H. Lemetre C. Aguilar R. R. Gordon E. A.. (2017). Commensal bacteria make GPCR ligands that mimic human signalling molecules. Nature 549, 48–53. 10.1038/nature23874 28854168 PMC5777231

[B10] Cotillard A. Kennedy S. P. Kong L. C. Prifti E. Pons N. Le Chatelier E.. (2013). Dietary intervention impact on gut microbial gene richness. Nature 500, 585–588. 10.1038/nature12480 23985875

[B11] Cox L. M. Yamanishi S. Sohn J. Alekseyenko A. V. Leung J. M. Cho I.. (2014). Altering the intestinal microbiota during a critical developmental window has lasting metabolic consequences. Cell 158, 705–721. 10.1016/j.cell.2014.05.052 25126780 PMC4134513

[B12] Derrien M. Vaughan E. E. Plugge C. M. de Vos W. M. (2004). *Akkermansia muciniphila* gen. nov., sp. nov., a human intestinal mucin-degrading bacterium. Int. J. Syst. Evol. Microbiol. 54, 1469–1476. 10.1099/ijs.0.02873-0 15388697

[B13] Devaraj S. Hemarajata P. Versalovic J. (2013). The human gut microbiome and body metabolism: implications for obesity and diabetes. Clin. Chem. 59, 617–628. 10.1373/clinchem.2012.187617 23401286 PMC3974587

[B14] Ding T. Schloss P. D. (2014). Dynamics and associations of microbial community types across the human body. Nature 509, 357–360. 10.1038/nature13178 24739969 PMC4139711

[B15] Falony G. Joossens M. Vieira-Silva S. Wang J. Darzi Y. Faust K.. (2016). Population-level analysis of gut microbiome variation. Science 352, 560–564. 10.1126/science.aad3503 27126039

[B16] Frayling T. M. Timpson N. J. Weedon M. N. Zeggini E. Freathy R. M. Lindgren C. M.. (2007). A common variant in the FTO gene is associated with body mass index and predisposes to childhood and adult obesity. Science 316, 889–894. 10.1126/science.1141634 17434869 PMC2646098

[B17] Gill S. R. Pop M. Deboy R. T. Eckburg P. B. Turnbaugh P. J. Samuel B. S.. (2006). Metagenomic analysis of the human distal gut microbiome. Science 312, 1355–1359. 10.1126/science.1124234 16741115 PMC3027896

[B18] Goodrich J. K. Waters J. L. Poole A. C. Sutter J. L. Koren O. Blekhman R.. (2014). Human genetics shape the gut microbiome. Cell 159, 789–799. 10.1016/j.cell.2014.09.053 25417156 PMC4255478

[B19] Hildebrandt M. A. Hoffmann C. Sherrill-Mix S. A. Keilbaugh S. A. Hamady M. Chen Y. Y.. (2009). High-fat diet determines the composition of the murine gut microbiome independently of obesity. Gastroenterology 137, 1716–1724.e1–2. 10.1053/j.gastro.2009.08.042 19706296 PMC2770164

[B20] Jensen L. J. Kuhn M. Stark M. Chaffron S. Creevey C. Muller J.. (2009). STRING 8–a global view on proteins and their functional interactions in 630 organisms. Nucleic Acids Res. 37, D412–D416. 10.1093/nar/gkn760 18940858 PMC2686466

[B21] Karlsson F. H. Tremaroli V. Nookaew I. Bergström G. Behre C. J. Fagerberg B.. (2013). Gut metagenome in european women with normal, impaired and diabetic glucose control. Nature 498, 99–103. 10.1038/nature12198 23719380

[B22] Khan M. T. Nieuwdorp M. Bäckhed F. (2014). Microbial modulation of insulin sensitivity. Cell Metab. 20, 753–760. 10.1016/j.cmet.2014.07.006 25176147

[B23] Kobyliak N. Conte C. Cammarota G. Haley A. P. Styriak I. Gaspar L.. (2016). Probiotics in prevention and treatment of obesity: a critical view. Nutr. Metab. 13:14. 10.1186/s12986-016-0067-0 26900391 PMC4761174

[B24] Kurokawa K. Itoh T. Kuwahara T. Oshima K. Toh H. Toyoda A.. (2007). Comparative metagenomics revealed commonly enriched gene sets in human gut microbiomes. DNA Res. 14, 169–181. 10.1093/dnares/dsm018 17916580 PMC2533590

[B25] Le Chatelier E. Nielsen T. Qin J. Prifti E. Hildebrand F. Falony G.. (2013). Richness of human gut microbiome correlates with metabolic markers. Nature 500, 541–546. 10.1038/nature12506 23985870

[B26] Li X. Shimizu Y. Kimura I. (2017). Gut microbial metabolite short-chain fatty acids and obesity. Biosci. Microbiota Food Health 36, 135–140. 10.12938/bmfh.17-010 29038768 PMC5633527

[B27] Liu R. Hong J. Xu X. Feng Q. Zhang D. Gu Y.. (2017). Gut microbiome and serum metabolome alterations in obesity and after weight-loss intervention. Nat. Med. 23, 859–868. 10.1038/nm.4358 28628112

[B28] Martens E. C. Koropatkin N. M. Smith T. J. Gordon J. I. (2009). Complex glycan catabolism by the human gut microbiota: the *Bacteroidetes* Sus-like paradigm. J. Biol. Chem. 284, 24673–24677. 10.1074/jbc.R109.022848 19553672 PMC2757170

[B29] Menni C. Zierer J. Pallister T. Jackson M. A. Long T. Mohney R. P.. (2017). Omega-3 fatty acids correlate with gut microbiome diversity and production of N-carbamylglutamate in middle aged and elderly women. Sci. Rep. 7:11079. 10.1038/s41598-017-10382-2 28894110 PMC5593975

[B30] Musso G. Gambino R. Cassader M. (2010). Obesity, diabetes, and gut microbiota: the hygiene hypothesis expanded? Diabetes Care 33, 2277–2284. 10.2337/dc10-0556 20876708 PMC2945175

[B31] Nicholson J. K. Holmes E. Kinross J. Burcelin R. Gibson G. Jia W.. (2012). Host-gut microbiota metabolic interactions. Science 336, 1262–1267. 10.1126/science.1223813 22674330

[B32] O'Hara A. M. Shanahan F. (2006). The gut flora as a forgotten organ. EMBO Rep. 7, 688–693. 10.1038/sj.embor.7400731 16819463 PMC1500832

[B33] Perry R. J. Peng L. Barry N. A. Cline G. W. Zhang D. Cardone R. L.. (2016). Acetate mediates a microbiome-brain-beta-cell axis to promote metabolic syndrome. Nature 534, 213–217. 10.1038/nature18309 27279214 PMC4922538

[B34] Qin J. Li R. Raes J. Arumugam M. Burgdorf K. S. Manichanh C.. (2010). A human gut microbial gene catalogue established by metagenomic sequencing. Nature 464, 59–65. 10.1038/nature08821 20203603 PMC3779803

[B35] Qin J. Li Y. Cai Z. Li S. Zhu J. Zhang F.. (2012). A metagenome-wide association study of gut microbiota in type 2 diabetes. Nature 490, 55–60. 10.1038/nature11450 23023125

[B36] Ridaura V. K. Faith J. J. Rey F. E. Cheng J. Duncan A. E. Kau A. L.. (2013). Gut microbiota from twins discordant for obesity modulate metabolism in mice. Science 341:1241214. 10.1126/science.1241214 24009397 PMC3829625

[B37] Rivière A. Selak M. Lantin D. Leroy F. De Vuyst L. (2016). Bifidobacteria and butyrate-producing colon bacteria: importance and strategies for their stimulation in the human gut. Front. Microbiol. 7:979. 10.3389/fmicb.2016.00979 27446020 PMC4923077

[B38] Rooks M. G. Garrett W. S. (2016). Gut microbiota, metabolites and host immunity. Nat. Rev. Immunol. 16, 341–352. 10.1038/nri.2016.42 27231050 PMC5541232

[B39] Rosenbaum M. Knight R. Leibel R. L. (2015). The gut microbiota in human energy homeostasis and obesity. Trends Endocrinol. Metab. 26, 493–501. 10.1016/j.tem.2015.07.002 26257300 PMC4862197

[B40] Samuel B. S. Shaito A. Motoike T. Rey F. E. Backhed F. Manchester J. K.. (2008). Effects of the gut microbiota on host adiposity are modulated by the short-chain fatty-acid binding G protein-coupled receptor, Gpr41. Proc. Natl. Acad. Sci. U.S.A. 105, 16767–16772. 10.1073/pnas.0808567105 18931303 PMC2569967

[B41] Scarpellini E. Campanale M. Leone D. Purchiaroni F. Vitale G. Lauritano E. C.. (2010). Gut microbiota and obesity. Intern. Emerg. Med. 5, 53–56. 10.1007/s11739-010-0450-1 20865475

[B42] Silverman M. Kua L. Tanca A. Pala M. Palomba A. Tanes C.. (2017). Protective major histocompatibility complex allele prevents type 1 diabetes by shaping the intestinal microbiota early in ontogeny. Proc. Natl. Acad. Sci. U.S.A. 114, 9671–9676. 10.1073/pnas.1712280114 28831005 PMC5594701

[B43] Suárez-Zamorano N. Fabbiano S. Chevalier C. Stojanović O. Colin D. J. Stojanović A.. (2015). Microbiota depletion promotes browning of white adipose tissue and reduces obesity. Nat. Med. 21, 1497–1501. 10.1038/nm.3994 26569380 PMC4675088

[B44] Thomas C. Gioiello A. Noriega L. Strehle A. Oury J. Rizzo G.. (2009). TGR5-mediated bile acid sensing controls glucose homeostasis. Cell Metab. 10, 167–177. 10.1016/j.cmet.2009.08.001 19723493 PMC2739652

[B45] Tilg H. Kaser A. (2011). Gut microbiome, obesity, and metabolic dysfunction. J. Clin. Invest. 121, 2126–2132. 10.1172/JCI58109 21633181 PMC3104783

[B46] Tolhurst G. Heffron H. Lam Y. S. Parker H. E. Habib A. M. Diakogiannaki E.. (2012). Short-chain fatty acids stimulate glucagon-like peptide-1 secretion via the G-protein-coupled receptor FFAR2. Diabetes 61, 364–371. 10.2337/db11-1019 22190648 PMC3266401

[B47] Turnbaugh P. J. Gordon J. I. (2009). The core gut microbiome, energy balance and obesity. J. Physiol. 587, 4153–4158. 10.1113/jphysiol.2009.174136 19491241 PMC2754355

[B48] Turnbaugh P. J. Hamady M. Yatsunenko T. Cantarel B. L. Duncan A. Ley R. E.. (2009). A core gut microbiome in obese and lean twins. Nature 457, 480–484. 10.1038/nature07540 19043404 PMC2677729

[B49] Turnbaugh P. J. Ley R. E. Mahowald M. A. Magrini V. Mardis E. R. Gordon J. I. (2006). An obesity-associated gut microbiome with increased capacity for energy harvest. Nature 444, 1027–1031. 10.1038/nature05414 17183312

[B50] Wright D. P. Rosendale D. I. Robertson A. M. (2000). Prevotella enzymes involved in mucin oligosaccharide degradation and evidence for a small operon of genes expressed during growth on mucin. FEMS Microbiol. Lett. 190, 73–79. 10.1111/j.1574-6968.2000.tb09265.x 10981693

[B51] Wu G. D. Chen J. Hoffmann C. Bittinger K. Chen Y. Y. Keilbaugh S. A.. (2011). Linking long-term dietary patterns with gut microbial enterotypes. Science 334, 105–108. 10.1126/science.1208344 21885731 PMC3368382

[B52] Zhao L. (2013). The gut microbiota and obesity: from correlation to causality. Nat. Rev. Microbiol. 11, 639–647. 10.1038/nrmicro3089 23912213

[B53] Zhernakova A. Kurilshikov A. Bonder M. J. Tigchelaar E. F. Schirmer M. Vatanen T.. (2016). Population-based metagenomics analysis reveals markers for gut microbiome composition and diversity. Science 352, 565–569. 10.1126/science.aad3369 27126040 PMC5240844

[B54] Zoetendal E. G. Rajilic-Stojanovic M. de Vos W. M. (2008). High-throughput diversity and functionality analysis of the gastrointestinal tract microbiota. Gut 57, 1605–1615. 10.1136/gut.2007.133603 18941009

